# Differential roles of the dorsal prefrontal and posterior parietal cortices in visual search: a TMS study

**DOI:** 10.1038/srep30300

**Published:** 2016-07-25

**Authors:** Yulong Yan, Rizhen Wei, Qian Zhang, Zhenlan Jin, Ling Li

**Affiliations:** 1 Key Laboratory for NeuroInformation of Ministry of Education, High-Field Magnetic Resonance Brain Imaging Key Laboratory of Sichuan Province, Center for Information in Medicine, School of Life Science and Technology, University of Electronic Science and Technology of China, Chengdu, 610054, China

## Abstract

Although previous studies have shown that fronto-parietal attentional networks play a crucial role in bottom-up and top-down processes, the relative contribution of the frontal and parietal cortices to these processes remains elusive. Here we used transcranial magnetic stimulation (TMS) to interfere with the activity of the right dorsal prefrontal cortex (DLPFC) or the right posterior parietal cortex (PPC), immediately prior to the onset of the visual search display. Participants searched a target defined by color and orientation in “pop-out” or “search” condition. Repetitive TMS was applied to either the right DLPFC or the right PPC on different days. Performance was evaluated at baseline (no TMS), during TMS, and after TMS (Post-session). RTs were prolonged when TMS was applied over the DLPFC in the search, but not in the pop-out condition, relative to the baseline session. In comparison, TMS over the PPC prolonged RTs in the pop-out condition, and when the target appeared in the left visual field for the search condition. Taken together these findings provide evidence for a differential role of DLPFC and PPC in the visual search, indicating that DLPFC has a specific involvement in the “search” condition, while PPC is mainly involved in detecting “pop-out” targets.

Brain mechanisms of attentional control play a central role in selecting relevant information as well as ignoring and suppressing irrelevant distractors. Visual attention is controlled by bottom-up sensory processes, as well as top-down cognitive processes^1^,^2^. Bottom-up control is an automatic process of response to salient stimuli^3^. Top-down control is a process which attempts to draw attention away from irrelevant distractors in order to focus on performing specific goals^4^,^5^,^6^,^7^.

Previous studies have proposed that a fronto-parietal attentional control network plays an important role in both types of attentional control^8^,^9^,^10^,^11^,^12^,^13^,^14^,^15^. For example, functional magnetic resonance imaging (fMRI) studies have suggested that the prefrontal cortex, the frontal eye field (FEF), the intraparietal sulcus, and the inferior parietal cortex form a frontal-parietal network for top-down attentional control^8^,^11^. In addition, transcranial magnetic stimulation (TMS) has been used to show that the right FEF^16^ and posterior parietal cortex (PPC)^17^ are critical for conjunction visual search.

The dorsolateral prefrontal cortex (DLPFC) is associated with working memory and is proposed to be the source of top-down bias^18^. To date there are only a few studies that have used TMS to examine the role of the frontal cortex in visual search. One of these studies showed that TMS applied over DLPFC impairs performance in a conjunction search task but not in feature search^19^. Similar results were also observed when TMS was applied over the FEF^20^. Overall these findings suggest that DLPFC, as well as FEF, are essential for the efficient performance of conjunction visual search.

In addition, the PPC, specifically the right PPC, has been identified as a primary component of the fronto-parietal network of spatial attention^8^. The findings regarding the role of the right PPC in visual search have been inconclusive. Some studies have shown that applying TMS over the PPC impairs performance in a conjunction search task but has no effect on a feature task^21^,^22^. Others have shown that TMS to the right PPC reduces the interference of a salient singleton distractor and facilitates visual search^23^. Furthermore, findings show that the disruption of the neural activity of this area interferes with attentional processes and results in contralateral neglect. For instance, the performance of participants was impaired in response to visual targets presented in the left visual field when TMS was applied over the right PPC^24^,^25^,^26^.

Studies in non-human primates give further support to the role of the fronto-parietal network in visual search. In one study non-human primates were required to find and focus on a visual target appearing among three distractors in separate “pop-out” and “search” conditions^27^. In the pop-out condition the distractors differed from the target in both orientation and color, such that the target drew attention automatically. Whereas in the search condition the target differed from the distractors only in orientation, so that the search required more effort. The findings showed that during the pop-out condition neurons in the lateral intraparietal area (LIP) responded to the target first, followed by the lateral prefrontal cortex (LPFC). We found support for these findings in a previous event-related potential (ERP) study^28^, in which participants were required to perform a similar task. The results provided evidence that the pop-out task was associated with greater activity over parietal areas and the search task was linked to greater activity over frontal areas.

In the present repetitive TMS study, we aimed to compare the contribution of the frontal and parietal cortices to the control of bottom-up and top-down visual attention. For this purpose, event-related TMS was applied either over the right DLPFC or the right PPC immediately prior to the onset of the visual search display. Participants performed a visual search paradigm, similar to the one used in our previous ERP study^28^. We hypothesized that, compared to the baseline session (no TMS), TMS over the right DLPFC would disturb performance in the search condition, while TMS to the right PPC would disturb the performance in the pop-out condition.

## Results

### Comparison of pre-TMS sessions

In order to ensure that the merger of the two pre-TMS sessions performed on each stimulation day can serve as the baseline, RTs and accuracies were compared, using a 2 (condition: pop-out vs. search) × 2 (session: pre-DLPFC, pre-PPC) × 2 (field: left vs. right) repeated measures ANOVA. Participants were faster in pop-out compared to search trials (*F*_(1, 15)_ = 142.99, *p* < 0.0001), and there was no significant difference in their performances between the two pre-TMS sessions (*F*_(1, 15)_ = 1.60, *p* = 0.23) nor between left and right visual fields (*F*_(1, 15)_ = 0.06, *p* = 0.82). All interactions were non-significant. Accuracy on pop-out trials was higher than that on search trials (*F*_(1, 15)_ = 77.23, *p* < 0.0001), but showed no other main effects or interactions (all *p* > 0.05). Thus, the average value of the two pre-TMS sessions was used as the baseline session. The mean of the RTs and accuracies for all the sessions are reported in Table 1.

### DLPFC-TMS effect

**Figure 1:**
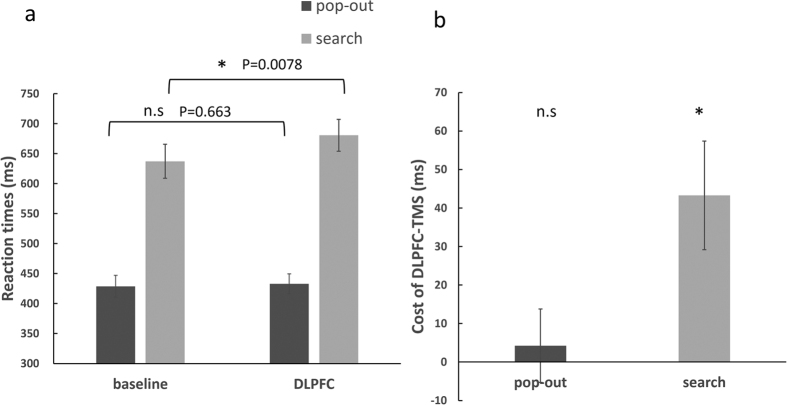
(**a**) The mean RTs of the pop-out and search conditions across baseline and DLPFC-TMS sessions. (**b**) The cost of DLPFC-TMS in the pop-out and search conditions. The error bars represent standard errors of the mean. An asterisk means *p* < 0.0083 (threshold). n.s = non significant.

RTs were compared using a repeated measures ANOVA, with condition (pop-out, search), DLPFC-TMS effect (baseline, DLPFC) and target visual field (left, right) as factors. There was a significant main effect for condition (*F*_(1, 15)_ = 151.55, *p* < 0.001), but the main effect of DLPFC-TMS was non-significant (*F*_(1, 15)_ = 4.40, *p* = 0.053). More importantly, there was an interaction between condition and DLPFC-TMS effect (*F*_(1, 15)_ = 18.48, *p* = 0.001). The other main effects and interactions were not significantly different (all *p* > 0.05). As there was no difference between the target visual fields, we merged the left and right field. A repeated measures ANOVA was conducted with condition (pop-out, search) and DLPFC-TMS effect (baseline, DLPFC) as factors. The results showed a significant main effect of condition (*F*_(1, 15)_ = 157.76, *p* < 0.001) and no significant effect of DLPFC-TMS (*F*_(1, 15)_ = 4.48, *p* = 0.052). A significant interaction between condition and DLPFC-TMS effect (*F*_(1, 15)_ = 19.91, *p* < 0.001) was found, indicating that the DLPFC-TMS effect was different between the two conditions (see Fig. 1a). To determine the source of this 2-way interaction, post hoc t-tests, Bonferroni corrected for multiple comparisons, were conducted to compare the baseline and the DLPFC-TMS session across the pop-out and search conditions. The threshold of the p-value for the 6 possible comparisons across the 4 conditions (4*3/2 = 6) was adjusted to 0.0083 (0.05/6 = 0.0083). The results showed that RTs were significantly prolonged in the DLPFC-TMS compared to the baseline session in the search condition (*t*_(15)_ = 3.06, *p* = 0.0078), but not in the pop-out condition (*t*_(15)_ = 0.44, *p* = 0.66). Figure 1b demonstrates the DLPFC-TMS cost, calculated by subtracting the baseline RT from the RT during the DLPFC-TMS session. As for accuracy, significant differences were observed between the pop-out and search conditions (*F*_(1, 15)_ = 77.23, *p* < 0.001), but no other main effects or interactions were found.

### PPC-TMS effect

**Figure 2:**
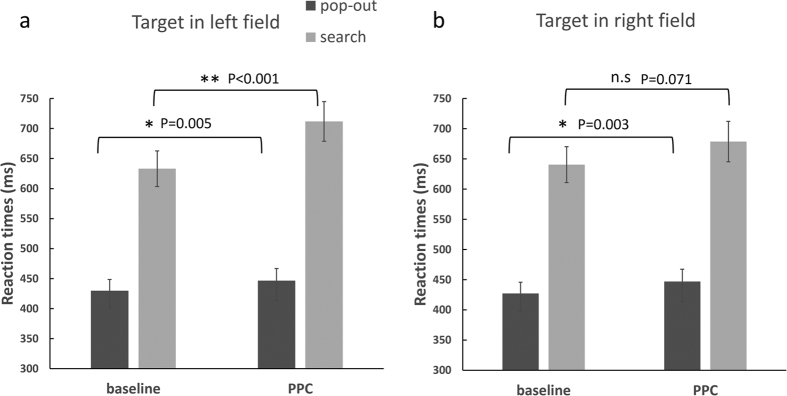
The mean RTs of the pop-out and search conditions across baseline and PPC-TMS sessions, when the target was presented in the left (**a**) and right (**b**) visual field. An asterisk means *p* < 0.0083 (threshold), and double asterisk means *p* < 0.001. n.s = non significant.

The comparison of the RTs between the baseline and the PPC-TMS session revealed faster performance in the pop-out compared to the search condition, (*F*_(1, 15)_ = 138.85, *p* < 0001), a significant main effect of PPC-TMS (*F*_(1, 15)_ = 19.63, *p* < 0.001) as well as significant interactions between condition × PPC-TMS (*F*_(1, 15)_ = 6.56, *p* = 0.02), PPC-TMS × field (*F*_(1, 15)_ = 5.12, *p* = 0.04) and between condition × PPC-TMS × field (*F*_(1, 15)_ = 10.28, *p* = 0.006). Other main effects and interactions were not found. Since there was a three-way interaction, we reassessed the PPC-TMS effect according to whether the target was presented in the left or right visual field. When the target was presented in the left field (see Fig. 2a), the ANOVA with condition (pop-out, search) and PPC-TMS effect (baseline, PPC) showed a significant main effect of condition (*F*_(1, 15)_ = 116.42, *p* < 0.001) and PPC-TMS (*F*_(1, 15)_ = 33.48, *p* < 0.001) as well as a significant condition × PPC-TMS interaction (*F*_(1, 11)_ =18, *p* = 0.001). Multiple comparisons showed that RTs during the PPC-TMS session were significantly slower than baseline in both the pop-out (*t*_(15)_ = 3.32, *p* = 0.005) and search conditions (*t*_(15)_ = 5.33, *p* < 0.001). When the target was presented in the right field, there were significant main effects of condition (*F*_(1, 15)_ = 132.56, *p* < 0.001) and PPC-TMS (*F*_(1, 15)_ = 7.24, *p* = 0.017), but the interaction was not significant (*F*_(1, 15)_ = 0.92, *p* = 0.35). Multiple comparisons showed that RTs during the PPC-TMS session were slower than that in the baseline session only in the pop-out (*t*_(15)_ = 3.56, *p* = 0.003), but not in the search condition (*t*_(15)_ = 1.94, *p* < 0.071) (see Fig. 2b).

**Figure 3:**
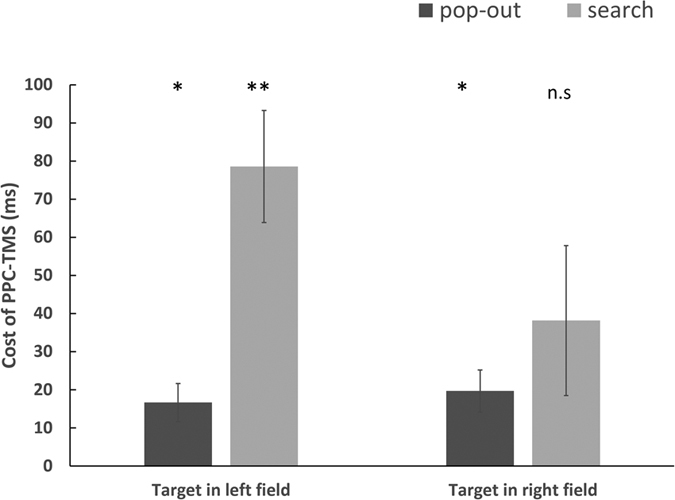
The cost of the PPC-TMS in the left and right visual field. An asterisk means *p* < 0.0083 (threshold), and double asterisk means *p* < 0.001. n.s = non significant.

To assess the difference between presentation of targets in the left and right visual fields, during the PPC-TMS session, we calculated the TMS cost across the visual fields (see Fig. 3). We compared TMS cost using a repeated measures ANOVA with condition (pop-out, search) and visual field (left, right) as factors. There was a significant main effect of condition (*F*_(1, 15)_ = 6.56, *p* = 0.02) and field (*F*_(1, 15)_ = 5.12, *p* = 0.039) as well as a significant condition × visual field interaction (*F*_(1, 15)_ = 10.28, *p* = 0.006). As for accuracy, significant differences were observed between the pop-out and search conditions (F_(1, 15)_ = 43.06, *p* < 0.001), but no other main effects or interactions were found.

### Vertex-TMS effect

The comparison of the RTs revealed a main effect for condition (*F*_(1, 15)_ = 195.04, *p* < 0.001) between the baseline and the Vertex-TMS session, showing faster RTs in the pop-out compared to the search condition. Other significant main effects and interactions were not found (all *p* > 0.05, specifically for the TMS effect: *F*_(1, 15)_ = 0.19, *p* = 0.67). As for the accuracy, there was a significant main effect for condition (*F*_(1, 15)_ = 72.27, *p* < 0.001) and for the visual field (*F*_(1, 15)_ = 7.59, *p* = 0.015), but no other significant main effects or interactions were found (all *p* > 0.05, specifically for the TMS effect: *F*_(1, 15)_ = 1.89, *p* = 0.19). The mean RTs and accuracies across the sessions are reported in Table 2.

### Time course of the TMS effect

To determine whether the TMS effect lasts we compared the RTs of the Pre-TMS and Post-session by performing a repeated measures ANOVA with condition (pop-out, search), time course (Pre-TMS, Post-session) and visual field (left, right) for DLPFC and PPC stimulation. There was a significant main effect of condition but no other main effects or interactions were found. These results indicate that there were no differences in the performance of the subjects between pre and 15 mins post TMS stimulation.

## Discussion

The objective of this study was to compare the contribution of frontal and parietal cortices to the control of bottom-up and top-down visual attention. We assessed the effects of DLPFC and PPC TMS stimulation on the performance of a visual search task. In this task, the findings showed that TMS applied over the DLPFC prolonged RTs in the search condition but not in the pop-out condition. On the other hand, PPC stimulation induced slower RTs in both the pop-out condition, and when the target was presented in the left visual field in the search condition.

We have shown the selective disruptive effect of DLPFC stimulation on the search condition but not the pop-out condition in a visual search task. DLPFC is associated with working memory^29^. The findings of the current study further suggest that this area has a role in guiding the performance during a visual search of a target, which is held in working memory. DLPFC may have a similar role to that of FEF, in the modulation of extra-striate responses to a visual stimulus^30^,^31^. In the search condition, the target differed from the distractors only in orientation, and thus top-down control during the visual search had a stronger frontal contribution^28^. It may be the case that DLPFC ‘holds’ a representation of the target and induces top-down biases to guide performance response to targets, while ignoring irrelevant distractors^32^. In addition, it has been suggested that DLPFC is involved when the task requires manipulation of multiple sources of information^33^. Thus, these memory processes as well as processes associated with the visual search of targets may have been responsible for the common activation of DLPFC^34^. Our findings further support this proposition since the search condition (selectively affected by DLPFC stimulation), requires cognitive coordination. In the pop-out condition, on the other hand, target localization was easier since the targets differed from the distractors in both orientation and color and participants could identify targets solely by color. As such DLPFC involvement was not necessary in this condition, as supported by our findings of a lack of DLPFC TMS effect in the pop-out condition.

Bottom-up attention signals may be first extracted in and relayed from the parietal cortex^35^. Our previous study has shown that the pop-out target localization generated a parietal maximal P300 component^28^. Furthermore, studies in primates^27^ have shown that during the pop-out condition neurons in the lateral intraparietal area (LIP) responded to the target faster than that in lateral prefrontal cortex (LPFC). These results suggest that bottom-up control during localization of the targets has a stronger parietal contribution. These findings are consistent with our current results showing that when TMS is applied over the right PPC, performance is disrupted in the pop-out condition. However, our findings are not in line with another study, which showed that PPC stimulation disrupts conjunction but not the following feature search^21^. The right PPC is primarily involved in processing spatial information. Thus, when location becomes critical for the successful completion of a feature search, the right PPC also becomes essential^36^. The present study required subjects to locate the target, and thus required the involvement of the PPC in the pop-out condition.

We also found that PPC stimulation prolonged RTs when the target was presented in the left visual field in the search condition. Several studies have shown differential TMS effects across visual fields. One study showed delayed RTs to target trials during stimulation of the right PPC relative to vertex stimulation^37^. This impairment of search performance was associated with TMS effects on the N2pc component over the right hemisphere^37^. PPC search deficits that were specific to the targets presented in the contralateral field were also observed in a conjunction search task^38^. Others have shown that the performance of participants is impaired when responding to visual targets presented in the left visual field, during application of TMS over the right PPC,^24^,^25^,^26^ and that right PPC stimulation produces a relative loss of top-down selection in the left hemi-field^39^. Together these findings suggest that the right PPC is not only involved in bottom-up process but also has a contribution to top-down processes. We propose that this area may serve as a hub that can modulate bottom-up signals and top-down biases in the visual search task.

The results of the control experiment show that several possible confounding, unspecific TMS effects can be excluded, such as, the distraction of the TMS coil on the participants head, the clicking noise of the device, or the presence of the experimenter standing behind the participant. The findings suggest that the unspecific TMS effects did not have a significant role in the participants’ performance and that the RT cost was induced by the actual TMS stimulation over DLPFC and PPC sites. Regarding the TMS stimulation time, we chose to deliver two pulses for a 200 ms time bin prior to the search display onset, for the following reasons. Firstly, other visual search studies have applied TMS shortly after the visual stimulus onset and have demonstrated DLPFC-TMS or PPC-TMS effects on visual search^19^,^22^. During the fixation phase, on the other hand, participants were required to sustain coherent target feature information and to prepare for the matching target, and thus our aim was to investigate the TMS effects for this phase. Secondly, applying TMS stimulation before the stimulus onset was reported in recent studies^17^,^40^, showing a decrease of the interference effects on search processing. However, when TMS is applied shortly after the visual stimulus onset, possibly overlapping with the N2pc ERP component (an indicator of spatial attention), unspecific TMS effects may influence the attentional processing directly.

Previous studies have shown the contributions of the DLPFC and PPC in saccade eye movements during spatial memory and attention^41^. Hence, one question that should be raised in the present study is whether the TMS effect could have been via saccadic eye movements. Other DLPFC-TMS and PPC-TMS studies have reported TMS effect on saccades^42^,^43^,^44^, although it may be difficult to differentiate the contribution related to saccades from that related to attention. In these experiments, the participants were required to make an initiative saccade to a location as accurately as possible, however, in our experiment participants were asked to centrally fixate throughout the recording and to press the corresponding key as quickly as possible to indicate whether the target was located to the left or right of the fixation. It is feasible that our participants may have performed passive saccadic eye movements towards the target. In general, on average we make two saccades when freely viewing 500 ms of visual stimuli, and the number of saccades may have been even lower under our experimental instructions of fixation, thus likely having little effect on the TMS results. Furthermore, another TMS study demonstrated that saccadic eye movements or blinks are performed in only a small portion of trials during a visual search task^19^.

In the present study we investigated the different contribution of the DLPFC and the PPC to bottom-up and top-down processes by applying TMS over these two sites. Our findings support the hypothesis that the right DLPFC has greater contribution to top-down control during the visual search task, while the right PPC has a role in bottom-up processes, but also in the modulation of top-down biases affecting performance in the contralateral visual search.

## Materials and Methods

### Participants

Sixteen subjects (six females, mean age ± SD = 20 ± 2 years) participated in the main experiment, and 16 subjects (six females, mean age = 22 ± 1 years) participated in the control experiment. There were four participants that overlapped and participated in both the main and the control experiments. There was no difference in the participants’ age and gender between the main experiment and the control experiment. All participants were right handed, had normal color vision and had no previous history of neurological problems. Standard exclusion criteria for TMS were applied: pregnancy, metallic implant, cardiac or neurological health condition, and specific medication. All participants gave written informed consent before the experiment and received monetary rewards after the experiment. The TMS session was performed according to the published safety guidelines^45^,^46^. All participants tolerated the TMS procedure well and did not report any adverse effects. The subjects were recruited from the University of Electronic Science and Technology of China. This study was approved by the local committee for the Protection of Human Subjects for the University of Electronic Science and Technology of China.

### Stimuli and Task

**Figure 4:**
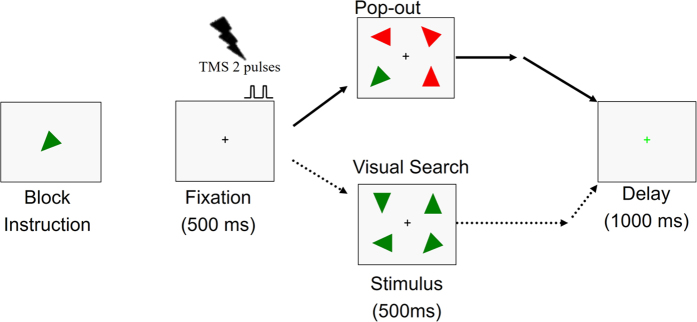
Experimental paradigm. The block instruction presented a target at the beginning of each block. A 500 ms fixation cross signaled the start of each trial and the TMS was applied during the last 200 ms of the fixation phase. The search display, composed of three distractors and the target, was presented for 500 ms. A green fixation cross then appeared on the screen for 1000 ms, indicating the end of the trial. Participants responded with a button press to indicate whether the target was on the left or right of the fixation cross.

The paradigm was modulated from a previous study^28^. Figure 4 illustrates an example of the stimulus sequence. The block instruction presented a target at the beginning of each block and never in the trial loops. The target was an isosceles triangle with a particular color (red or green) and orientation (one of eight). Participants were required to remember the color and orientation. A 500 ms fixation cross signaled the start of each trial and two TMS pulses were applied during the last 200 ms of the fixation phase. The first and second pulse was triggered 300 ms and 400 ms after the fixation onset, respectively. Then the stimulus display appeared consisting of the target and three distractors in the four quadrants of the screen (upper-left, lower-left, upper-right, and lower-right). The target was randomly located in one of the quadrants. The distractors were selected to create either a “pop-out” or a “search” condition. In the pop-out condition the distractors differed from the target in both color and orientation, allowing for bottom-up capture of attention. In the search condition only the orientation of the distractors differed from that of the target, calling upon more controlled, top-down mechanisms of attention. The two conditions were randomly mixed and each condition was presented on 50% of the trials. Half of the targets were presented in left visual field and half were presented in the right visual field. Participants were required to maintain fixation on the central cross throughout the experimental trial and to identify as quickly and as accurately as possible on which side of the screen the target appeared. It is noteworthy that participants were explicitly requested to maintain fixation on the central cross instead of searching one side of the search display. Subjects used their right hand to press button 1 for targets appearing on the left side of the screen and button 2 for targets appearing on the right side, regardless of whether the targets appeared on the upper or lower halves of the screen. The stimuli were displayed for 500 ms, regardless of whether the participants responded or not. Finally, a green fixation cross appeared on the screen for 1000 ms, indicating the end of the trial. Participants were able to make a decision during the stimulus display or delay period.

Stimuli, viewed at a distance of 60 cm, were presented on a DELL monitor with a resolution of 1024 × 768 pixels and a refresh rate of 60 Hz. The center of each triangle was 6.2 cm. vertically, either up or down, from the center and 8.2 cm. horizontally, either right or left, from the center, at a visual angle of 5.34° from fixation. E-prime (Psychology Software Tools, Pittsburgh, PA) was used for stimulus presentation, recording of the behavioral results, and generation of the TMS triggering.

### Experimental procedures

**Figure 5:**
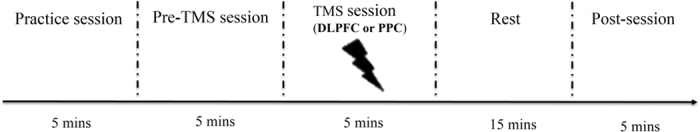
Schematic illustration of the timeline of the sessions.

In each participant TMS was applied over two sites, the right DLPFC and the right PPC, each on a different day. The order of the two sites was counterbalanced across all the participants. Each session consisted of two blocks and a total of 128 trials, with 64 trials for the pop-out condition and 64 trials for the search condition. Each session lasted approximately 5 mins. On each of the experimental days, participants first performed a practice session to ensure that they were familiar with the task. Then, participants performed a session without TMS stimulation, which we termed the pre-TMS session, followed by the TMS session (DLPFC or PPC). Finally, after a period of 15 minutes rest, to allow for the TMS effect to wash out, participants performed a session without TMS stimulation (post-session). The timeline of the procedures is illustrated in Fig. 5.

In order to exclude unspecific TMS effects, a control experiment was performed in another group of subjects. Participants performed the practice, baseline and TMS sessions, with TMS applied to the vertex.

### TMS and stimulation sites

TMS was applied using a Magstim super rapid magnetic stimulator and an air-cooled figure-of-eight coil having an outer winding diameter of 70 mm (Magstim Company Limited, Whiteland, UK). Two pulses of 10 Hz were delivered during the last 200 ms of the fixation phase, and the first and second pulses were triggered 300 ms and 400 ms after the fixation onset, respectively. A total of 256 pulses (128*2) were applied in each session. Stimulation intensity was set at 100% of each participant’s individually determined motor threshold. The motor threshold was defined as the lowest intensity of single-pulse TMS stimulation required to evoke motor potentials of at least 50 μv in 5 out of 10 trials, in the contralateral first dorsal interosseous (FDI) muscle, following stimulation over the hand area of the participant’s right motor cortex. The average motor threshold values were 51 ± 5.6% of the stimulator’s max output power.

**Figure 6:**
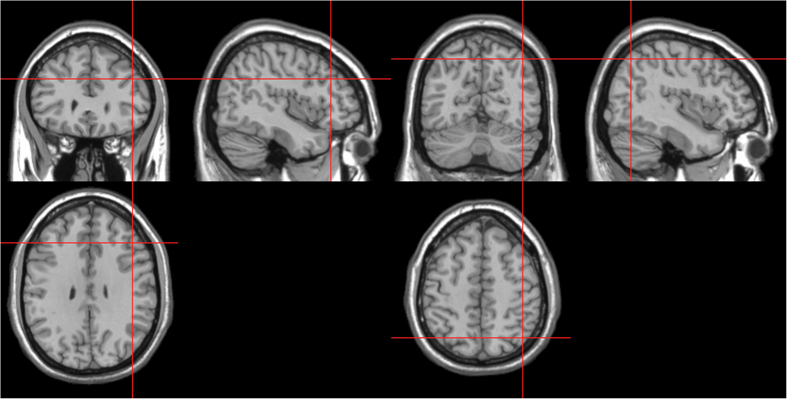
Coronal, axial, sagittal views of the stimulated site right-DLPFC (left), MNI coordinates: 45, 30, 31 and right- PPC (right), MNI coordinates: 43, −65, 51, depicted on a standard template from MRIcro.

TMS Stimulation sites were localized in individual participants using a frameless stereotaxy system (BrainSight Frameless, Rogue Research, Montreal, Canada). Landmarks on the participants’ head were co-registered to a standard MRI template using this system. Stimulation sites were defined using coordinates from previous TMS studies^39^,^47^. MNI coordinates for right DLPFC were [45 30 31] (SD of 5, 4, 4 mm) and for right PPC were [43–65 51] (SD of 3, 4, 3 mm) (Fig. 6). These coordinates were then used as stimulation targets and the TMS coil was placed on the corresponding location over the participants’ scalp. Brainsight was used to track the position of the TMS coil throughout the stimulation period, ensuring that it remained on the target location. For the TMS stimulation over the vertex (control experiment), this was localized as a midpoint between the inion and the nasion and equidistant from the left and right ear.

### Data analysis

Reaction times (RTs) and correct response rates (accuracy) were measured. Trials were rejected if they had an incorrect response or lacked a button press between 200 and 1200 ms after the onset of the stimulus array. A repeated measures ANOVA was performed to compare RTs and accuracies, with condition (pop-out vs. search), TMS effect (baseline vs. DLPFC or PPC or vertex) and the visual field of target presentation (left vs. right) as within-subject factors. If necessary, baseline and TMS session were further compared using post hoc t-tests, Bonferroni corrected for multiple comparisons. The threshold of the p-value for the 6 possible comparisons across the 4 conditions (4*3/2 = 6) was adjusted to 0.0083 (0.05/6 = 0.0083).

## Additional Information

**How to cite this article**: Yan, Y. *et al*. Differential roles of the dorsal prefrontal and posterior parietal cortices in visual search: a TMS study. *Sci. Rep.* **6**, 30300; doi: 10.1038/srep30300 (2016).

## Figures and Tables

**Table 1:** Mean values (and s.e.m) of RTs and accuracies for the main experiment.

Session	Target Field	Pop-out condition		Search condition	
		RTs (ms)	Accuracy (%)	RTs (ms)	Accuracy (%)
Baseline	left	430 (18)	99.0 (0.4)	633 (30)	90.8 (2.0)
	right	427 (18)	99.4 (0.2)	641 (30)	87.1 (1.6)
DLPFC-TMS	left	434 (16)	99.0 (0.3)	668 (27)	86.6 (2.2)
	right	432 (17)	99.2 (0.3)	673 (30)	87.8 (2.1)
Post of DLPFC	left	424 (14)	99.2 (0.3)	630 (31)	90.8 (1.9)
	right	425 (16)	99.0 (0.4)	661 (29)	85.9 (1.7)
PPC-TMS	left	447 (20)	99.8 (0.3)	712 (33)	86.3 (3.0)
	right	447 (20)	98.5 (0.5)	697 (34)	87.3 (2.5)
Post of PPC	left	417 (14)	99.6 (0.3)	636 (33)	91.3 (2.0)
	right	413 (13)	98.7 (0.6)	642 (32)	88.1 (1.9)

Note: Baseline was calculated by averaging the value of two days’ pre-TMS sessions.

**Table 2:** Mean values (and s.e.m) of RTs and accuracies for the control experiment.

Session	Target Field	Pop-out condition		Search condition	
		RTs (ms)	Accuracy (%)	RTs (ms)	Accuracy (%)
Baseline	left	463 (17)	99.6 (0.3)	713 (32)	88.0 (2.0)
	right	459 (16)	98.5 (0.5)	704 (30)	86.1 (2.1)
Vertex-TMS	left	445 (10)	99.6 (0.3)	696 (28)	91.9 (1.5)
	right	455 (13)	97.9 (0.7)	710 (29)	86.8 (2.1)
